# Waste-to-energy technology selection and capacity planning in a multi-facility waste management system

**DOI:** 10.1007/s43937-026-00164-1

**Published:** 2026-06-07

**Authors:** Ahmed Saif, Khaled Zoroufchi Benis

**Affiliations:** 1 Department of Industrial Engineering, Dalhousie University, 5269 Morris Street, Halifax, NS B3H 4R2 Canada; 2 Department of Process Engineering and Applied Science, Dalhousie University, 1360 Barrington Street, Halifax, NS B3J 1Z1 Canada

**Keywords:** Waste-to-energy, Location-allocation, Technology selection, Capacity planning, Environment

## Abstract

Waste-to-energy (WtE) technologies provide a viable solution for dealing with the large quantities of municipal solid waste (MSW) generated in modern societies. However, they must be optimally integrated with waste management and energy distribution networks to maximize their economic and environmental benefits. A strategic optimization model is proposed for locating waste processing facilities, allocating MSW-generating communities to them, determining the types and capacities of equipment in each, and directing the flow of energy products (electricity, heat, and hydrogen) from them to demand nodes, aiming to achieve two objectives: maximizing profit and minimizing greenhouse gas emissions. The cost, emission, and conversion functions of three WtE technology alternatives (TAs) are meticulously derived so they can be incorporated into the mathematical model. The proposed approach is applied to a realistic case study in Nova Scotia, Canada. Results show that the type and location of WtE technologies depend on the objective sought, the cost structure of TAs, and the price of energy products. Plasma arc gasification with hydrogen separation is found to be the most economically viable WtE technology due to the high value of hydrogen it produces, whereas anaerobic digestion with combined heat and power has the lowest carbon footprint. Sensitivity analysis revealed that the optimal WtE system configuration is quite sensitive to the price of hydrogen, while the technology capacity lower limit barely changes the system’s economic and environmental performance. Conversely, variations in waste composition and subsidy policies caused no change in the profit-maximizing system configuration.

## Introduction

Effective management of municipal solid waste (MSW) represents a major environmental challenge for policymakers nowadays [1]. On the one hand, around one-third of the approximately 2 billion tons of MSW generated each year globally are not being adequately managed from an environmental standpoint [2]. On the other hand, collecting, transporting, sorting, and disposing of these vast quantities of MSW consumes a significant amount of energy and generates substantial amounts of greenhouse gas (GHG) emissions [3]. Recent advances in Waste-to-Energy (WtE) technologies like incineration, anaerobic digestion (AD), pyrolysis, and gasification offer a promising solution to the MSW problem faced by communities worldwide. This is why some agencies, such as the United States Environmental Protection Agency (USEPA), have listed MSW as a renewable energy source that can be used to attain the Sustainable Development Goals (SDGs) [4].

Nevertheless, successful integration of WtE solutions within existing energy systems requires a thorough assessment of both the technologies themselves and the networks used to carry their inputs (i.e., waste) and outputs (i.e., energy products). Indeed, large, centralized WtE processing facilities enjoy a cost advantage over small ones due to economies of scale and improved technical efficiency. However, they are also associated with higher costs and GHG emissions for shipping MSW from communities and energy products to demand points [5]. Furthermore, the technical, economic, and environmental feasibility of each WtE technology depends on the quantity and composition of the MSW it utilizes. Hence, it is necessary to jointly consider the WtE technology selection, location, and capacity planning, and the configuration of waste collection and energy distribution networks when assessing the viability of utilizing WtE solutions in energy systems.

Despite the growing researchers’ interest in optimizing waste collection (see, e.g., [6–8]) and WtE plant location and technology selection (see, e.g., [9, 10]), little attention has been paid to addressing the two problems simultaneously. Among the few studies that consider this combination is the two-stage stochastic optimization model proposed in reference [11], which optimizes the location of WtE plants and the shipment of MSW to them under uncertainty in waste composition. However, the issues of technology selection and capacity planning were overlooked in this work, assuming they were exogenously predetermined. Conversely, in works that focused on the latter issues, e.g., [10], the spatial aspects of waste management, i.e., collection and transportation of MSW, were ignored.

Within a relevant stream of research, Esfilar et al. [12] studied the technoeconomic feasibility of both a standalone WtE gasification process and a hybrid process that combines it with renewable resources to generate electricity and heat using MSW from a university campus and neighbouring areas. Similarly, but at a larger scale, Armoo et al. [13] designed and assessed the feasibility of a hybrid WtE facility in Accra, Ghana, that integrates solar PV, AD and pyrolysis to treat unsegregated waste to generate electricity, hydrogen, or bio-compressed natural gas, in addition to a suite of by-products. These studies, among numerous others, while valuable, employed a simulation approach that assessed a small number of pre-determined alternatives, unlike the optimization framework proposed in the current work. The advantage of using hybrid WtE systems that combine multiple technologies, versus relying on a single technology, was underscored by Inayat et al. [14], motivating the modeling approach in this paper.

The two studies that are found to be most closely related to the current one are those of Gonçalves Mollica et al. [15] and Saatchi et al. [16]. A profit maximization model to integrate gasification and hybrid incineration into a landfill-based waste management system in Brazil is proposed in the former reference, with waste transportation between existing landfills to improve energy generation efficiency and financial income. In contrast to the current work, which employs a high-level modeling approach for WtE technologies and aims to balance economic and environmental objectives, a more granular thermodynamic equilibrium model is used in reference [15], and the focus was on economic performance only. In reference [16], a two-phase decision-making framework is introduced, by which the WtE technologies are selected in the first phase based on multiple criteria, including economic, environmental, and social risks, while the second phase involves optimizing the MSW supply chain (i.e., generation, separation, processing, and disposal) under uncertainty. Conversely, the methodology proposed in the current work decides the WtE technology selection, location, and capacity, and the MSW and energy flows simultaneously, not sequentially.

### Contributions

To fill the afore-described gaps, the paper makes the following contributions:

1. It proposes a novel, high-level, strategic optimization model for designing an integrated MSW-to-energy (MSWtE) system that can process the MSW generated in a given geographical region. Unlike previous studies that had a narrow focus on certain aspects of the system or made the decisions sequentially, the proposed model jointly addresses the interconnected decisions of locating waste processing facilities and determining the types and capacities of WtE technologies in them, the assignment of MSW-generating communities to facilities, and the flow of energy products (electricity, heat, and hydrogen) to demand points. The proposed bi-objective model aims to maximize the total revenue from selling energy products, net of MSW and energy products’ shipping costs and facilities’ capital and operational costs, and minimize the total GHG emissions from the entire process.
2. It derives, based on elementary technical and publicly available data, the cost, emission, and conversion functions for three technology alternatives (TAs) that can be installed in WtE processing facilities. The considered TAs are: 1) incineration with a steam power generator, 2) AD with combined heat and power (CHP) units, and 3) plasma arc gasification (PAG) with hydrogen separation. The derived expressions are rigorous, yet simple enough to be used in the MSWtE optimization model. Given that equipment cost functions are concave, the formulation results in a nonconvex optimization problem that can be handled using modern global optimization solvers.
3. It applies the proposed approach to a realistic case study in the province of Nova Scotia (NS), Canada, to design an MSWtE system that can supply electricity, heat, and hydrogen while reducing landfilling. Sensitivity analysis is conducted to quantify the impact of changing key model parameters, and the results are thoroughly analyzed to extract managerial and policy insights.

It is worth noting that the current paper significantly extends a previous conference proceeding by the first author [17].

### Paper organization

The remainder of this paper is organized as follows. The next section briefly describes the problem and its mathematical formulation. The cost, emission, and conversion functions of the different TAs are meticulously derived in Sect. 3 such that they can be incorporated into the mathematical model. Section 4 describes the case study and the results obtained based on it, in addition to the sensitivity analysis conducted and the insights drawn, whereas a conclusion is presented in Sect. 5.

## Problem description and formulation

This section begins by providing a formal description of the considered problem, followed by its modeling assumptions and notations, and finally, the proposed mathematical formulation.

### Problem description

The decision-maker in the problem under consideration is an entity responsible for collecting and managing MSW in a given geographical area (e.g., city, province, country). This area is subdivided into zones/communities, indexed by *i∈ I*. Currently, MSW is collected from these zones and sent to landfills. However, to reduce the negative environmental impacts associated with landfilling and generate some revenue, the decision-maker is contemplating utilizing some combinations of WtE and energy conversion technologies. These combinations, referred to hereinafter as technology alternatives, are indexed by *t∈ T*. Each TA takes MSW as an input and generates one or more energy products, e.g., electricity, heat or hydrogen, indexed by *p∈ P*. For example, a TA can consist of an incinerator that burns waste to generate heat, combined with a steam power generator that converts heat to electricity, which is then sold to potential customers. The list of TAs and their outputs is provided in the next subsection. TA equipment is to be installed in processing facilities (or, simply, facilities) that can be located in a subset of predetermined potential locations indexed by *j∈ J*. These locations are typically selected based on environmental and logistical criteria, i.e., being sufficiently distant from population centers to avoid negative impacts, but accessible by waste trucks and close enough to energy sinks. Moreover, demand points are indexed by *k∈ K*, and each energy product can be shipped/transmitted from the facilities to one or more of these sinks. Examples of sinks include downstream processing facilities, residential communities, and industrial complexes. Therefore, the MSWtE system can be represented as the digraph $G(\mathcal {N},\mathcal {A})$, where $\mathcal {N}=I\cup J\cup K$ encompasses the nodes of this graph and $\mathcal {A}$ is the set of arcs connecting these nodes.

The problem aims to determine the optimal MSWtE system configuration, which entails the locations of processing facilities, the types and capacities of TA equipment to be installed in each facility, the flow of MSW from zones to facilities, and the flow of the energy products from them to demand nodes. Two objectives are sought: maximizing monetary profit and minimizing GHG emissions. The profit to be maximized is the difference between the products’ sales revenue and the total cost of the system, which includes the waste collection cost, the TAs’ capital and operating costs, and the costs associated with delivering products to sinks and the residual waste (if any) to landfills. Since waste management is considered a “cost center” by municipalities, the optimal profit can be negative, i.e., the system cost can be greater than the revenue generated. On the other hand, GHG emissions include those emitted in transporting MSW to facilities, processing the MSW, and delivering end products to sinks.

### Modeling assumptions

The following assumptions are used to formulate the problem:

1. The quantity and composition of the MSW collected from each zone are known and fixed throughout the planning horizon. This is a reasonable assumption given that the model deals with the steady-state, long-term operation of the system and does not consider seasonal fluctuations. This assumption enables the problem to be formulated as a single-period, infinite-horizon optimization model.
2. MSW originating from each zone is assigned to a single processing facility. This assumption aligns with the current practices in waste management and simplifies the calculation of waste collection costs and emissions.
3. The cost and GHG emissions for collecting the MSW from a given zone and sending it to a given facility can be computed externally, e.g., by solving a vehicle routing problem, and used as input in the model. In other words, the issue of optimal routing is beyond the scope of this model.
4. An installed piece of equipment will be operated continuously and steadily at its rated capacity. Thus, the rated capacities of WtE equipment are calculated based on the quantities of MSW to be processed by them.

It is worth noting that these assumptions, while justifiable and realistic, could be easily relaxed by making minor adjustments to the mathematical model, allowing it to accommodate alternative scenarios. For example, related to the first assumption, seasonal fluctuations in MSW quantity or composition can be incorporated by adding an index for season to the operational variables and parameters. In contrast, uncertainties in these parameters can be handled using stochastic, robust, or fuzzy optimization methods, depending on the type of uncertainty (epistemic or aleatory), the availability of distributional information, and the risk attitude of the decision-maker. Likewise, Assumption 2 could be relaxed by using continuous allocation decision variables signifying the fraction of a zone’s MSW output assigned to each processing facility. As for Assumption 3, the model can be augmented by introducing standard vehicle routing decision variables and constraints and linking them to the location and flow variables. Finally, if equipment capacities must be selected from predetermined values or if a modular system is sought, the continuous capacity variables can be replaced by binary or integer variables representing, respectively, whether a specific equipment size is selected or the number of units to be installed, thus relaxing Assumption 4.

### Notations

To formulate the problem, we introduce the binary decision variables $x_{ij}$ and $y_{jt}$ to denote, respectively, whether the MSW originating from zone *i* is processed in facility *j* (i.e., the facility opened in location *j*, if any), and whether TA *t* is used in it. In addition, the continuous variable $q_{jt}$ denotes the installed capacity of TA *t* in facility *j*. For consistency, the installed capacity of all equipment is measured in terms of the MSW mass they can process per unit time (ton/year). We also introduce the continuous flow variable $z_{jkp}$, which represents the quantity of product *p* shipped/transmitted from facility *j* to sink *k*.

Each TA has cost and emission functions, $c_t(q_{jt})$ and $e_t (q_{jt})$, respectively, which map the TA capacity installed in the facility to its annual cost and the quantity generated of GHG emissions from its continuous operation. Moreover, TA *t* supplies product *p* according to the conversion function $f_{tp}(q_{jt})$. This function maps the annual quantity of MSW processed by the TA, which equals its installed capacity, to the amount of product delivered. The annual quantity of MSW generated in zone *i* is $w_i$, whereas the cost and GHG emissions resulting from collecting this quantity and sending it to facility *j* are $a_{ij}$ and $b_{ij}$, respectively. A unit of product *p* can be sold in demand/sink node *k* for $s_{kp}$, and it costs $d_{jkp}$ to ship/transmit a unit of product *p* from facility *j* to sink *k*, whereas the GHG emissions associated with this shipment is $h_{jkp}$. Furthermore, if TA *t* is used in a facility, there are upper and lower limits ($\overline{q}_t$ and $\underline{q}_t$, respectively) on its installed capacity. The set of feasible arcs for shipping product *p* is denoted by $\mathcal {A}_p\subset \mathcal {A}$.

### Mathematical formulation

Based on the aforementioned problem description, modeling assumptions, and notations, the problem can be formulated as follows:

1 $$\begin{aligned}&\text {maximize}\; & TP=\sum _{j\in J}\sum _{k\in K}\sum _{p\in P}\left( s_{kp}-d_{jkp}\right) z_{jkp}-\sum _{j\in J}\sum _{t\in T}c_t(q_{jt})-\sum _{i\in I}\sum _{j\in J}a_{ij}x_{ij}; \end{aligned}$$

2 $$\begin{aligned}&\text {minimize}\; & TE=\sum _{j\in J}\sum _{t\in T}e_t(q_{jt})+\sum _{i\in I}\sum _{j\in J}b_{ij}x_{ij}+\sum _{j\in J}\sum _{k\in K}\sum _{p\in P}h_{jkp}z_{jkp}; \end{aligned}$$

3 $$\begin{aligned}&\text {subject to:}\nonumber \\ & &\sum _{j\in J}x_{ij}=1,\qquad \forall i\in I; \end{aligned}$$

4 $$\begin{aligned} & &\sum _{i\in I}w_{i}x_{ij}=\sum _{t\in T}q_{jt},\qquad \forall j\in J; \end{aligned}$$

5 $$\begin{aligned} & &\sum _{t\in T}f_{tp}(q_{jt})=\sum _{k\in K}z_{jkp},\qquad \forall j\in J,p\in P; \end{aligned}$$

6 $$\begin{aligned} & &\underline{q}_t y_{jt}\le q_{jt} \le \overline{q}_t y_{jt},\qquad \forall j\in J, t\in T; \end{aligned}$$

7 $$\begin{aligned} & &z_{jkp}=0,\qquad \forall (j,k)\notin \mathcal {A}_p; \end{aligned}$$

8 $$\begin{aligned} & &x_{ij},y_{jt}\in \{0,1\},\qquad \forall i\in I, j\in J, t\in T; \end{aligned}$$

9 $$\begin{aligned} & &z_{jkt}\ge 0,\qquad \forall j\in J, k\in K, p\in P. \end{aligned}$$

The three terms of the profit maximization objective function defined by Eq. (1) represent the revenue from selling products (net of shipping cost), the TA capacity costs, and the MSW collection and shipping costs, respectively. The GHG emissions minimization objective function represented by Eq. (2) consists of the emissions generated by the TAs, and the emissions related to the shipping of MSW and energy products. The first constraint modeled by Eq. (3) stipulates that each waste-collection zone is assigned to a single processing facility. Equation (4) ensures that the installed capacity of equipment is sufficient to process all MSW allocated to the processing facility, whereas Eq. (5) ensures the quantity shipped equals the quantity generated for each facility and product type. The limits on installed capacity are enforced by the constraint formulated using inequality (6). Equation (7) prevents any shipment of products when it is not technically feasible, e.g., when the distance prevents it, or there is no demand for the product, while expressions (8 and 9) are variable domain constraints. The class of this multi-objective optimization model depends on the functions $c_t(q_{jt})$, $e_t(q_{jt})$, and $f_{tp}(q_{jt})$, since all other terms are linear. The next section discusses their functional forms.

## System characteristics

In this section, the TAs are defined, and their cost, emission, and conversion functions are derived based on publicly available data. Finally, the costs and emissions corresponding to the shipment of MSW to facilities, and of energy products from them to demand nodes, are estimated.

### Technology alternatives

**Fig. 1:**
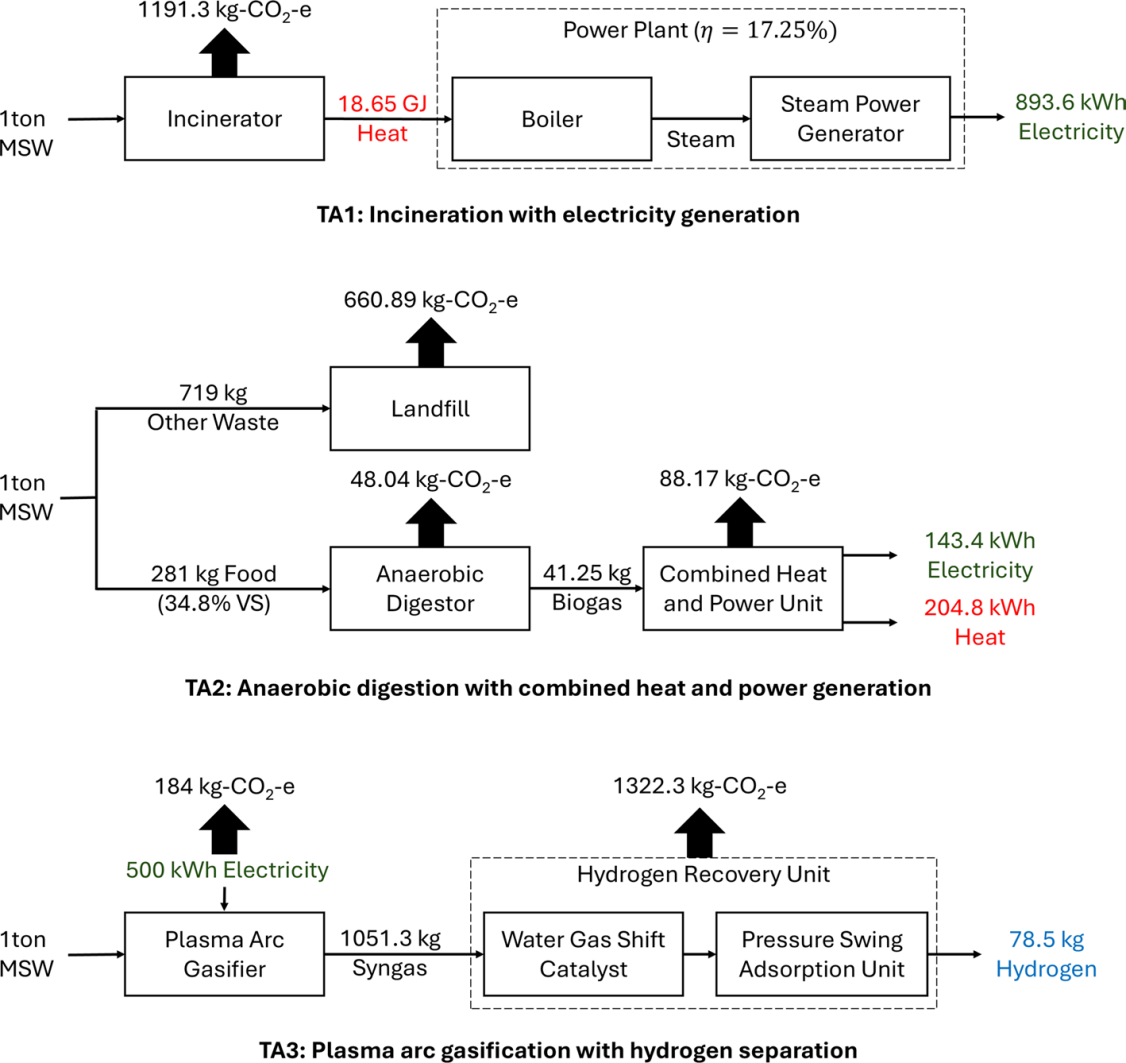
A simplified graphical representation of the technology alternatives considered in this study

Three TAs, illustrated in Fig. 1, are considered for installation in processing facilities:

[TA1]: Incineration with a steam power generator: the heat generated by incinerating MSW is used to boil water into steam that turns a turbine to generate electricity [18].

[TA2]: Anaerobic digestion with combined heat and power units: a series of biological processes in the absence of oxygen convert organic waste to biogas, which is then used in a CHP unit to generate heat and electricity [19].

[TA3]: Plasma arc gasification with hydrogen separation: PAG is an extension of conventional gasification, wherein plasma torches are used to substantially raise the temperature of MSW in an oxygen-starved environment to generate synthetic gas (syngas). The syngas is then purified before being sent to a hydrogen recovery system that consists of a water gas shift (WGS) unit to convert the carbon monoxide to hydrogen and a Pressure Swing Adsorption (PSA) unit for the separation and purification of hydrogen [20].

### Cost, emission, and conversion functions of TAs

The total cost of each piece of equipment includes both capital (acquisition, commissioning, installation) costs and operation and maintenance (O&M) costs, whereas only the direct (operational) GHG emissions are considered, but no embedded emissions. Table 1 depicts the cost, emission, and conversion functions of the different TAs. A detailed description of how these functions are derived is outlined next.

**Table 1:** The cost, emission, and conversion functions for the technology alternatives considered in this study

TA	$$c_t(q_{jt})$$ ($/year)	$$e_t(q_{jt})$$ (kg-CO_2_e/year)	$$f_{tp}(q_{jt})$$
1	$$1,691.9q_{j1}^{0.7753}+256.4q_{j1}^{0.8594}$$	$$1,191.3q_{j1}$$	$$893.6q_{1j}$$ kWh-e
2	$$95.7q_{j2}+66.2q_{j2}^{0.667}$$	$$797.1q_{j2}$$	$$143.4q_{j2}$$ kWh-e
			$$204.8q_{j2}$$ kWh-h
3	$$188.9q_{j3}+81.8q_{j3}^{0.8111}$$	$$1,506.3q_{j3}$$	$$78.5q_{j3}$$ kg-H_2_

#### Technology alternative 1

**Table 2:** MSW composition

Waste Type	Fraction (%)	Carbon (%)	Hydrogen (%)	Oxygen (%)	Moisture (%)
Food	28.1	51	5	39	65.2
Paper	12.3	45	5	45	6.9
Plastic	20.2	56	6	26	0.3
Textile	11.6	59	6	19	7.8
Gross	72.2	38.0	3.9	24.0	20.1

The cost function of the first TA (*t=1*), which consists of an incinerator, a boiler, and a steam power generator, is based on reference [21]. The investment cost in $M for the plant is estimated at $$2.3507Cap_{INC}^{0.7753}$$, where $$Cap_{INC}$$ is the plant capacity in 1000 metric tons per year. We first change the units in this equation to $’s (by multiplying the constant by $$10^6$$) and tons of MSW/year (by dividing $$Cap_{INC}$$ by 1000), and adjust for inflation (3% per year for 9 years) to obtain an estimated initial capital cost of $$\$14,482q_{j1}^{0.7753}$$. This capital cost is then amortized while assuming a useful life of 15 years, a discount rate of 8%, and no salvage value, i.e., using a capital recovery factor (CRF) of 0.11683. Thus, the capital cost function becomes $$1691.9q_{j1}^{0.7753}$$$/year. The annual O&M cost according to reference [21] is $$0.0744Cap_{INC}^{0.8594}$$, which is similarly adjusted to $$256.4q_{j1}^{0.8594}$$$/year after changing the units and accounting for inflation.

CO_2_ emissions from incinerating the waste depend on the carbon content of the MSW, its moisture content, and the oxidation factor, according to the IPCC GLs mass balance method explained in reference [22]. Table 2 shows the breakdown of different organic materials in the MSW and the corresponding mass ratios of carbon, hydrogen, and oxygen, as estimated in reference [10] for a landfill in NS. Based on 38.0% carbon content, 20.1% moisture content (since the remaining components have virtually no moisture), and an oxidization factor of 1 (the default value), each ton of MSW generates 1191.3 kg of CO_2_.

Next, the annual electricity generated from TA1 is computed as $\eta _{inc}. LHV_{MSW}. q_{j1}$, where $\eta _{inc}$ is the efficiency of the incineration power plant, estimated in reference [21] using the T-S diagram at 17.25%. On the other hand, $LHV_{MSW}$, the lower heating value of MSW, is calculated in reference [23] as:

$$ LHV_{MSW}=0.0041868(1+0.15[O])\left( 7,837.667[C]+33,888.889[ht]-\frac{[O]}{8}\right) , $$

where [*O*], [*ht*] and [*C*] are the oxygen, hydrogen, and carbon contents in the MSW, respectively. Based on this formula, the LHV of MSW equals 18.65 GJ/ton. This value is close to those estimated in reference [24] using different methods. Thus, the incineration plant generates 893.6 kWh of electricity per ton of MSW.

#### Technology alternative 2

The second TA (*t = 2*) includes an Anaerobic digester and a CHP unit. The capital cost for a new AD facility in Canada that produces biogas is estimated in reference [25] to be $630/ton of annual processing capacity, whereas the operating cost is estimated to be $80/ton of feedstock. A wide range of cost estimates has been provided in reference [26], depending on the feedstock type, end product, specific AD technology, and facility capacity and location. However, the above estimates seem reasonable and will be used herein. First, we adjust these values for inflation (3% per year for 14 years). Upon amortizing the capital cost using a CRF of 0.11683 (similar to the first alternative), the total annual cost per ton of feedstock amounts to $232.34/ton. It should be noted, however, that only the organic (food) part of the MSW (28.1%*× *1000=281 kg/ton of MSW) can be used for AD, whereas the remainder (719 kg/ton of MSW) ends up in a landfill for a tipping fee of $100/ton, a typical rate in North America, amounting to 71.9 $/ton of MSW.

The biogas yield from organic waste ranges between 0.3 and 0.5 $$m^3$$/kg volatile solids (VS) [27]. The VS fraction in MSW depends on its composition, but can be reasonably taken as the fraction of food waste after subtracting moisture, i.e., 28.1%*× *(1 - 0.652) = 9.78%, or 97.8 kg/ton of MSW. Hence, the AD total annual cost per ton of MSW is $232.34*× *97.8/1000=$22.72. Furthermore, assuming a mid-point yield of 0.4 $$m^3$$/kg VS, each ton of MSW generates about 0.4*× *97.8=39.1 $$m^3$$ (1598.8 moles at 1 atmosphere of pressure and 25$$^{\circ }$$C, given that a mole of gas at these conditions occupies 0.024466 $$m^3$$) of biogas. This quantity is equivalent to 41.25 kg of biogas that has a molecular weight of 25.8 (assuming a composition of 35% CO_2_, which has a molecular weight of 44, and 65% CH_4_, which has a molecular weight of 16). Although the solid residues resulting as byproducts of AD might have some industrial and agricultural uses, we disregard them here and focus on the energy products. Based on the aforementioned composition of the generated biogas, its LHV is 65% of that of methane (55MJ/kg), amounting to 35.75 MJ/kg, or equivalently 41.25*× *35.75=1474.7 MJ per ton of MSW.

Next, it is necessary to compute the capacity of the CHP unit that uses the generated biogas to model its energy outputs and costs. For this, the input energy per year, $1,474,700q_{j2}$ kJ, is divided by (365 *× * 24 *× * 60 *× * 60) to obtain around $0.04676q_{j2}$ kW power input. We assume a constant electrical efficiency of 35% and a constant heat efficiency of 50% for the CHP unit. These values are quite typical in the literature (see, e.g., [28]). Thus, the CHP unit shall have a rated (electrical) capacity of $35\%\times 0.04676q_{j2}=0.01637q_{j2}$ kW and deliver $50\%\times 0.04676q_{j2}=0.02338q_{j2}$ kW of heat. In terms of energy delivery per year, these power rates correspond to $8,760\times 0.01637q_{j2}=143.4q_{j2}$ kWh of electricity and $8,760\times 0.02338q_{j2}=204.8q_{j2}$ kWh of heat, which define the conversion function.

The investment cost function of a sparkplug ignition (SI) biogas-fueled CHP unit is estimated to be $$4,639Cap_{CHP}^{-0.333}$$ Euros/kW, where $$Cap_{CHP}$$ is the rated capacity in kW, in 2005 [28]. Accounting for inflation and the currency exchange rate (1.08181 $/Euro), the investment cost is estimated to be $$8,800(0.01637q_{j2})^{1-0.333}= \$566.6q_{j2}^{0.667}$$. Again, amortizing the capital cost over the same useful life yields an annual cost of approximately $$66.2q_{j2}^{0.667}$$. On the other hand, the O&M cost is estimated in reference [29] to be 4 Euros/MWh in 2007, which is equivalent to $1.06/ton of MSW after accounting for inflation and exchange rate. Hence, the cost function becomes: $$(22.72+71.9+1.06) q_{j2}+66.2q_{j2}^{0.667}\approxeq 95.7q_{j2}+66.2q_{j2}^{0.667}$$, where the linear terms represent, respectively, the AD, landfilling and CHP O&M costs, whereas the concave term represents the CHP capital cost.

The GHG emissions for TA2 have three main sources. First, some biogas escapes during the AD process, estimated in reference [30] as 6.3% of the total production. Based on the aforementioned biogas composition, each ton of MSW generates 35%*× *41.25=14.44 kg of CO_2_ and 65%*× *41.25=26.81 kg of CH_4_. The 100-year global warming potential (GWP) of methane is 27.9 [31]. Thus, the CO_2_-equivalent emissions from this source are 6.3%*× *(14.44+27.9*× *26.81)=48.04 kg/ton of MSW. The second source of GHG emissions is the combustion of biogas in the CHP unit to generate electricity and heat. In particular, one mole of methane generates one mole of carbon dioxide according to CH_4_ + 2O_2_ *→ * CO_2_ + 2 H_2_O, or equivalently, every 16 grams of CH_4_ generates 44 grams of CO_2_. Thus, 26.81 kg/ton of methane generates (44/16)*× *26.81=73.73 kg/ton of CO_2_, which is added to the remaining CO_2_ in biogas to yield a total of 73.73+14.44=88.17 kg/ton of MSW from the second source. The third source corresponds to landfilling the remaining (non-organic) part of the MSW. Landfills are notorious for generating large amounts of GHGs, especially methane and carbon dioxide. Besides the organic waste used in the AD process, the other primary degradable waste type is paper, which has a carbon content of 12.3%*× *45%*× *1000=55.35 kg/ton of MSW (see Table 2). According to [32], the dissimilation factor of biogenic carbon as landfill gas is estimated at 0.5, besides another 0.04 as leachate, totaling 0.54, which is the fraction of carbon that leaves the landfill as gas within 100 years, amounting to 0.54*× *55.35=29.89 kg/ton of MSW. It is estimated that 55% of this carbon becomes CH_4_ and 45% becomes CO_2_ [32]. Thus, each ton of MSW processed using TA2 generates 55%*× *29.89*× *(16/12)=21.92 kg of CH_4_ and 45%*× *29.89*× *(44/12)=49.32 kg of CO_2_, or equivalently, 27.9*× *21.92+49.32=660.89 kg-CO_2_e. Adding this quantity to the emissions from the first two sources, the emission function is $(48.04+88.17+660.89)q_{j2}=797.1q_{j2}$ kg-CO_2_e/year.

#### Technology alternative 3

The third TA (*t = 3*) includes a PAG unit that uses heat to generate syngas from MSW and a PSA unit that recovers hydrogen from syngas. The syngas generated using PAG is typically composed (by volume) of 42.9% CO, 34.8% H_2_, and 16.6% CO_2_, with the remaining volume consisting of Nitrogen and traces of CH_4_ [33]. These fractions correspond to approximately 57.8% CO, 3.35% H_2_, and 35.1% CO_2_ by weight, with a carbon percentage of (12/28)*× *57.8%+(12/44)*× *35.1%=34.34% in the syngas. The quantity and composition of the syngas depend on the equivalence ratio, reactor temperature, waste composition and physical properties, and composition and inlet temperature of the gasifying medium [34]. However, for simplicity, we assume that these operating parameters are predetermined and fixed. Assuming a carbon conversion efficiency (CCE) of 95% and that MSW is composed of 38% carbon (see Table 2), out of each ton of MSW, 95%*× *38%*× *1000=361 kg of carbon is converted to syngas. Since syngas consists of 34.34% carbon, approximately 361/34.34%=1,051.3 kg of syngas is generated per ton of MSW. Based on the aforementioned composition (by mass) of syngas, this quantity contains approximately 606.7 kg CO, 35.2 kg H_2_ and 368.9 kg CO_2_. Given LHVs of 10.112 MJ/kg and 120.96 MJ/kg for CO and H_2_, respectively, the calorific value of the generated syngas quantity per ton of MSW is (606.7*× *10.112+35.2*× *120.96)/1000=10.393 GJ, which represents 55.73% of the LHV for MSW (in GJ/ton). This value is within the 50–80% typical cold gas efficiency values for gasification as reported in reference [35]. The carbon monoxide is then converted to hydrogen in the hydrogen recovery system according to CO + H_2_O *→ * CO_2_ + H_2_, i.e., each 28 kg of CO generates 44 kg of CO_2_ and 2 kg of H_2_, assuming perfect conversion. Thus, 606.7*× *(2/28)=43.3 kg H_2_ and 606.7*× *(44/28)=953.4 kg CO_2_ are generated from this conversion. With that, the total quantities of H_2_ and CO_2_ generated per ton of MSW are 35.2+43.3=78.5 kg and 368.9+953.4=1,322.3 kg, respectively.

PAG is a relatively new technology with only a few pilot and small-scale projects completed. It is, therefore, quite difficult to extract the technology cost function from the few data points available. We rely on the 6 projects reported in reference [20] and perform power regression for the relationship between capital cost and capacity (in tons of MSW per year) to obtain the relation $$$491.11q_{j3}^{0.8111}$$. By accounting for inflation (3% per year over 12 years) and amortizing the capital cost using the CRF of 0.11683, we get the investment cost function for the plasma reactor as $$81.8q_{j3}^{0.8111}$$ $/year. As for the O&M costs, we use the estimate of $86.5/ton made in reference [35]. As for the hydrogen recovery process, Mostafavi Sani et al. [10] estimated the cost to be $26,712/MWh of energy output from the PSA. Given that hydrogen has a LHV of 120.96/3.6=33.6 kWh/kg, the cost is (26,712/1000)/33.6=0.795 $/kg-H_2_, or equivalently 0.795*× *78.5=62.4 $/ton of MSW.

Besides the CAPEX and OPEX, the PAG technology uses significant quantities of electricity to generate heat. In reference [34], it is estimated that PAG consumes between 1,200 and 2,500 MJ (333.3 and 694.4 kWh) of electricity per ton of MSW, whereas reference [36] estimated that for a large-scale (3,600 MW) plasma gasification power plant, the electricity consumption is 115 kWh per ton of MSW for the reactor only. Assuming the almost-midrange electricity consumption of 1,800 MJ/ton (i.e., 500 kWh/ton), and assuming a cost of 8 cents per kWh (the NS rate for industrial consumers), the electricity cost is 0.08*× *500=40 $/ton of MSW. The GHG emissions associated with electricity consumption depend on the power generation mix. Assuming GHG emissions of 368 kg-CO_2_ per MWh (the 2023 US average), each ton of MSW generates (500/1000)*× *368=184 kg of CO_2_ from electricity consumption.

### Shipping costs and emissions

The costs and GHG emissions associated with the collection of MSW from zones and shipping them to processing facilities are considered first. Given that these facilities are usually located far away from residential communities to protect people from their negative health and environmental effects [11], it is reasonable to separate the processes of collecting and shipping the MSW. The waste collection process is performed at each community independently, and its economic and environmental costs are not dependent on the facility to which the waste is shipped. Hence, these costs can be computed offline, e.g., by solving a routing problem, irrespective of the allocation of zones to facilities. On the other hand, the shipping costs depend on the quantities shipped and the distance traveled. Since the MSW amounts collected from any community are vast relative to the capacity of a single waste truck, it can be assumed that shipping costs and emissions are linearly proportional to the MSW quantity. Likewise, the shipping economic and environmental costs are linearly proportional to the distance between the community and the facility. Given that the capacity of a typical garbage truck is 18 tons, that it consumes an average of 0.5 liters of diesel per km [37], and that the price of diesel is around 1.2 $/liter, the fuel cost is 0.5*× *1.2/18=0.033 $/km/ton. Overheads (e.g., driver, maintenance) of 50% are then added to reach an MSW shipping cost of 0.05 $/km/ton. A diesel engine generates around 2.7 kg-CO_2_/liter; thus, the emissions from MSW shipping are 0.5*× *2.7/18=0.075 kg-CO_2_/km/ton. Note that these values must be multiplied by 2 to account for the round trip.

Hydrogen is transported in tube trailers having a capacity of 1,042 kg. Using the same parameters for the truck as above, the hydrogen shipping cost is around 1.73 $/km/ton, and the corresponding CO_2_ emission is 2.59 kg-CO_2_/km/ton (both ways). The transmission costs of electricity (heat) include both the capital cost of the transmission line (pipe) and the transmission losses. Both of these costs are assumed to be proportional to the distance. The cost per kilometer of power transmission lines varies widely depending on voltage level, terrain, land ownership (i.e., right-of-way), technology (i.e., overhead vs. underground), and substation interconnections. For instance, a recent interconnection feasibility study in NS estimated the cost of a 44-km, 230 kV transmission line that connects a 300 MW wind farm to the grid at $60.72 millions, or, equivalently, $1.38 million per km [38]. A lower estimate of around $832,000 for a 69 kV transmission line that spans one km was reported in a 2010 feasibility study [39]. So, accounting for inflation, the former estimate seems reasonable. Likewise, heat transmission pipes vary in cost depending on many factors. A recent feasibility study of a mine thermal energy storage in Glace Bay, NS, estimated the cost per km of a 107 km dual-pipe network to range between $0.8 and $1.2 million [40].

In contrast, the carbon emissions are calculated based on the transmission losses only. Hydrogen is assumed to be sold for 3 $/kg, which is a quite typical price of “grey” hydrogen in 2025, whereas electricity is sold to the utility at $0.175/kWh, the feed-in tariff in NS for electricity generated using CHP from biomass. Estimating the selling price of heat is a challenging task since it depends on the application. However, considering that most of it will be used in district heating to substitute heat generated by 90%-efficiency furnaces that use propane (LHV=7.02 kWh/liter, bulk price=0.79 $/liter), it is reasonable to assume a selling price of 0.79/(7.02*× *90%)=0.125 $/kWh. This price is close to the 0.111 $/kWh (30.74 $/GJ) estimated levelized cost of heat (LCOH) for waste heat in NS reported in reference [40], which represents about 22% reduction compared to the 0.142 $/kWh 2025 high-cost benchmark for oil heating in Halifax, NS [41].

## Numerical results

The numerical results obtained based on a realistic case study are reported and analyzed in this section.

**Fig. 2:**
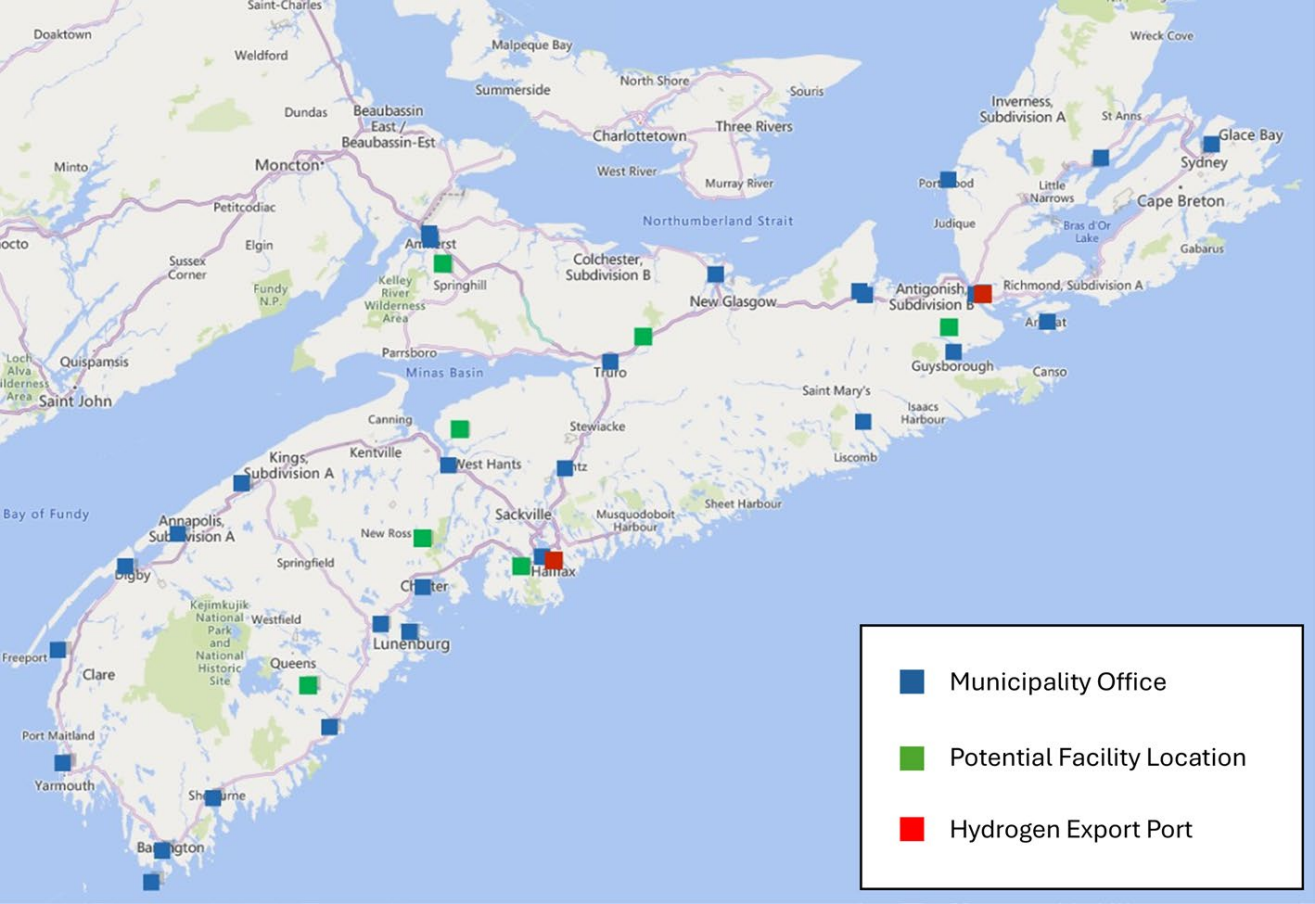
NS municipality offices, landfills, and hydrogen ports

### Case study

The proposed approach is implemented to design an MSWtE system in NS. Figure 2 shows the map of NS, with the locations of municipality offices, landfills (i.e., potential facility locations), and hydrogen export ports marked. The residential MSW (i.e., garbage) quantities for each of NS’s 29 municipalities between April 2022 and March 2023 (the latest available data) are extracted from reference [42], totaling around 120 thousand tons. As for potential locations for the processing facilities, we utilize the existing seven MSW disposal sites in NS [43]. The shortest driving distance between each municipal office/town hall and potential facility location is found using Google Maps and used to calculate MSW shipping costs and emissions.

**Table 3:** A list of NS municipalities, their annual MSW quantities (in tons), and their approximate one-way driving distances to potential facility locations (in kilometers, rounded to the closest integer)

ID	Municipality	Quantity	Distance to Facility						
			1	2	3	4	5	6	7
1	CBRM	13,853.30	289	400	179	413	395	472	534
2	Inverness County	2,153.00	196	308	87	321	303	374	436
3	Port Hawkesbury	383.67	156	267	46	280	262	334	395
4	Richmond County	966.93	199	300	89	324	305	377	439
5	Victoria County	1,091.00	236	337	127	361	343	414	476
6	Antigonish County	2,260.64	100	200	46	224	206	278	340
7	Antigonish Town	440.79	98	198	49	223	205	276	338
8	Guysborough	1,126.17	159	258	15	284	265	370	399
9	Mulgrave	172.68	153	253	29	278	155	331	393
10	Pictou	4,565.59	44	127	120	174	155	227	289
11	St Marys	555.79	129	229	86	212	236	284	342
12	Amherst	975.05	121	17	253	207	188	260	322
13	Colchester County	5,350.23	18	101	160	106	89	130	221
14	East Hants	4,210.95	75	148	216	55	63	108	170
15	Cumberland County	2,703.51	119	15	250	204	186	263	319
16	HRM	48,350.55	122	195	260	12	78	80	142
17	Annapolis County	1,646.00	292	366	434	203	168	168	94
18	Valley Waste	5,552.00	253	327	395	171	129	132	125
19	Barrington	2,113.01	352	433	479	230	254	195	114
20	Chester	2,629.31	178	251	304	56	84	25	87
21	Clarkes Harbour	310.45	372	453	498	250	274	215	134
22	MJSB	4,679.78	208	281	334	86	110	51	53
23	Shelburne	1,158.16	320	393	447	198	222	163	82
24	Queens	2,594.04	262	335	388	140	164	105	21
25	Lunenburg	374.14	212	285	338	90	114	55	66
26	West Hants	2,297.14	125	198	266	71	27	38	133
27	Clare	1,339.44	360	433	460	261	236	226	152
28	Digby	2,564.00	319	392	460	220	195	185	111
29	Yarmouth County	3,737.00	410	483	552	292	195	185	176

Table 3 depicts the MSW quantities and the travel distances used to compute the model parameters. Since all facilities are currently connected to the electricity grid, the cost associated with delivering the generated electricity is set to zero. On the other hand, heat is sent to the closest community within 30 km of the facility for district heating. A penalty of 2% per km of the heat selling price is assumed to account for losses and infrastructure cost (e.g., pipes). GHG emissions for electricity and heat transmission are assumed to be negligible. As for hydrogen, it is shipped to the closest of the ports of Halifax or Point Tupper, the two ports that can be used to export hydrogen in NS. To avoid unrealistic solutions, if a TA is used at any location, its installed capacity cannot be less than 5,000 tons/year of MSW. This lower bound on the installed capacity is imposed to ensure that the optimizer does not select configurations that are technically impractical, such as a micro-scale (*< 1* MW) steam turbine, or economically infeasible due to the strong economies of scale in all TAs and the other fixed overheads (e.g., labor) not fully captured in the cost functions, which provide a good approximation only over some range of capacities.

### Base-case results

**Table 4:** Optimal solutions and values obtained for the base case scenario with three objectives: profit maximization, emission minimization, and a combination thereof

Facility	TA1	TA2	TA3	Zones
*Profit maximization:*	*TP = 2,731.16*			*TE = 183.23*
3	-	-	23,004	1–9, 11
4	-	-	97,150	10, 12–29
*Emission minimization:*	*TP = -7,421.82*			*TE = 96.88*
1	-	9,916	-	10, 13
2	-	5,059		3, 7, 11, 12, 15
3	-	21,624	-	1, 2, 4–6, 8, 9
4	-	52,562		14, 16
5	-	7,849	-	18, 26
6	-	7,309	-	20, 22, 25
7	-	15,152	-	17, 19, 21, 23, 24, 27–29
*Bi-objective:*	*TP = -2,166.14*			*TE = 140.05*
1	-	9,916	-	10, 13
2	-	5,049	-	7, 11, 12, 15, 25
3	-	22,007	-	1–6, 8, 9
4	-	7,258	60,462	14, 16, 18, 20, 22, 26
7	-	15,462	-	17, 19, 21, 23, 24, 27–29

The table shows total profit (TP) in thousand $, total emissions (TE) in kiloton CO_2_e, and the processing capacities of installed TAs in thousand tons of MSW, along with the zones assigned to each facility

The problem is solved using Gurobi 12.0.0, called via the gurobipy library from Python 3.9. Table 4 depicts the results for three scenarios, namely the single-objective profit maximization and emission minimization cases, and a bi-objective case that balances the two objectives. The well-known epsilon-constraint method is used in the bi-objective scenario, by which profit maximization is considered the primary objective, whereas the quantity of GHG emissions is capped at its mid-point value (i.e., 140.051 kiloton CO_2_e) between the optimal values of the two single-objective models. The table shows the installed capacity of each TA in each facility, the assignment of zones to facilities, and the total profit (TP) and total emissions (TE).

**Fig. 3:**
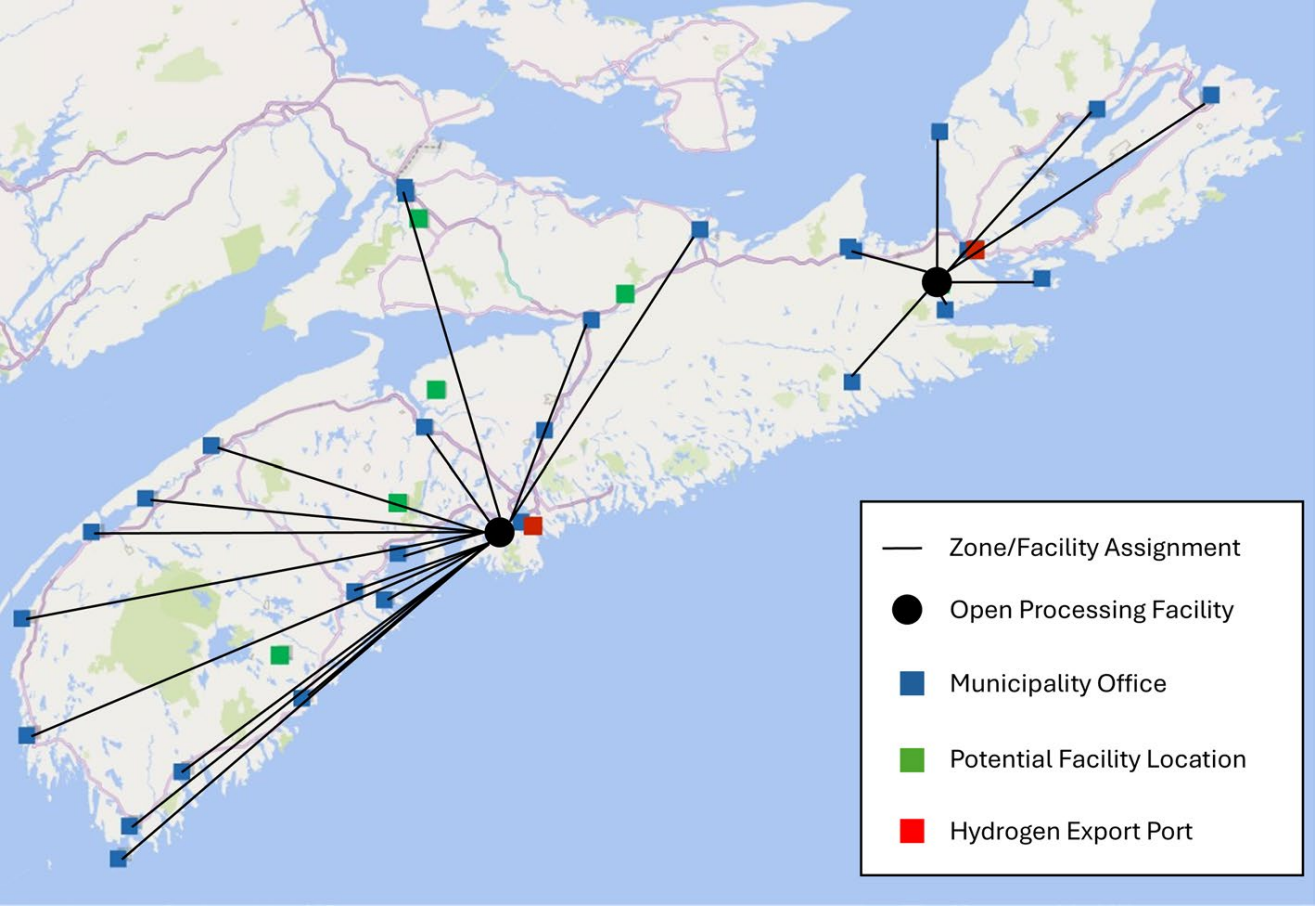
Optimal MSWtE system configuration for the profit maximization objective

Figure 3 shows the optimal system configuration for the profit maximization objective. Notably, only two processing facilities (in Guysborough and Halifax) are opened, and TA3 (PAG with hydrogen separation) is used in both. This result reflects both the impact of economies of scale, which favors large, centralized facilities, and the high revenue from hydrogen sales compared to electricity and heat. Both facilities are very close to the hydrogen exportation ports to minimize the relatively high hydrogen shipping costs. It is interesting to note that this solution generates a positive profit of around $22.73 and about 1.525 ton-CO_2_e per ton of MSW.

**Fig. 4:**
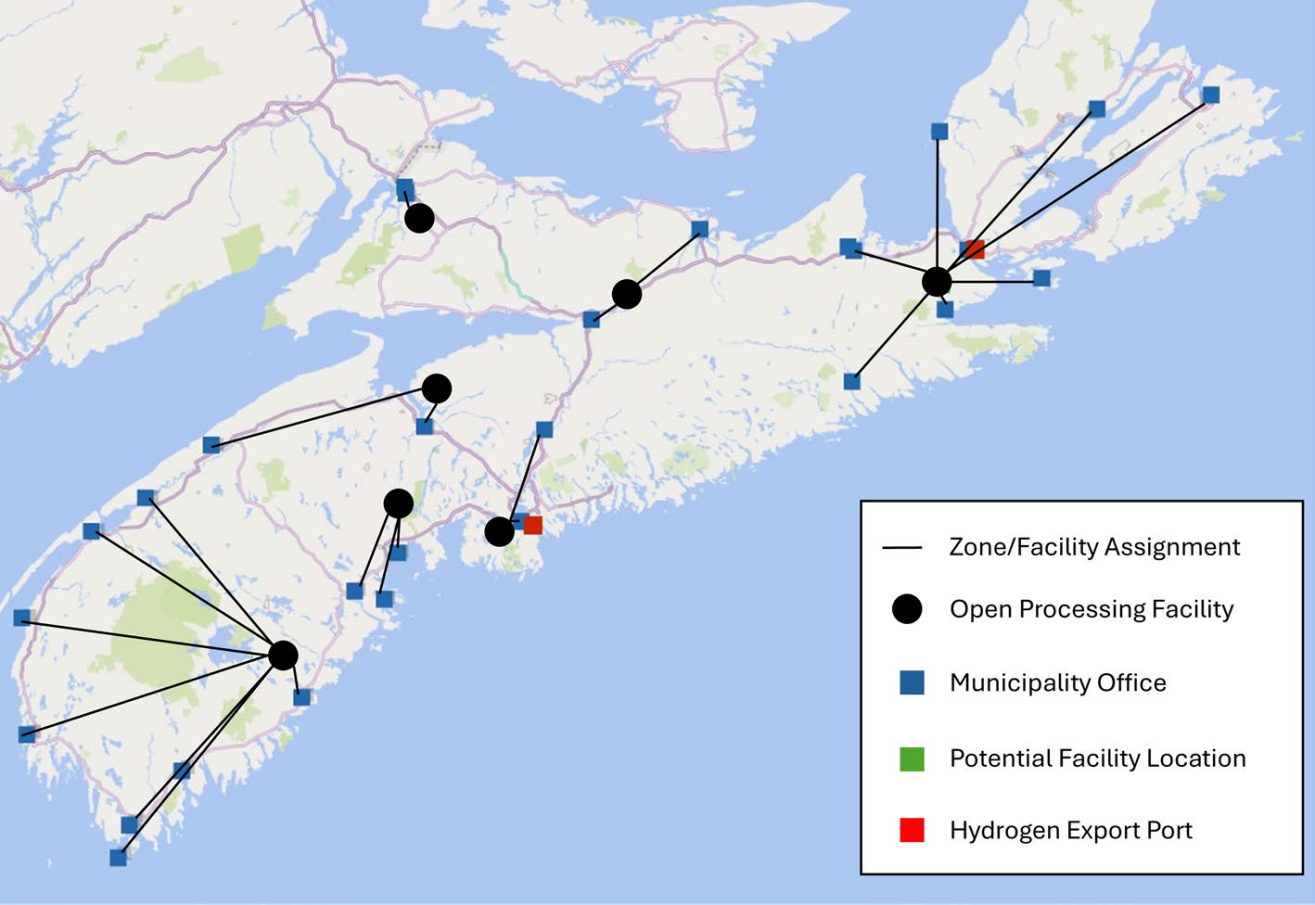
Optimal MSWtE system configuration for the emission minimization case

In contrast, as shown in Fig. 4, the emission minimization objective led to a decentralized MSWtE system with all 7 potential facilities opened, and each municipality is assigned to the closest facility to minimize shipping-related GHG emissions, while respecting the minimum allowable capacity constraint. Since the emission functions for all TAs are linear in processing capacity, there is no value in consolidation. Furthermore, TA2 (AD with CHP) is used in all facilities since it has the lowest per unit amount of GHG emissions. The emissions generated per ton of MSW have decreased to 0.806 ton-CO_2_e, but at a monetary loss of $61.77/ton of MSW. In the bi-objective case, which tries to balance economic and environmental considerations, five facilities are opened in Colchester, Cumberland, Guysborough, Halifax, and Queens. Four of these facilities use TA2 exclusively, whereas the Halifax facility (which is close to a hydrogen port) combines TA2 and TA3 to generate electricity, heat, and hydrogen. This scenario results in a relatively modest loss of $18.03 and GHG emissions of 1.166 ton-CO_2_e per ton of MSW. Interestingly, TA1 (incineration) is not used under any scenario due to the high cost and emissions of the steam power plant, which becomes economically viable only with a large processing capacity ($$q_{j1}>300,000$$ ton/year).

**Fig. 5:**
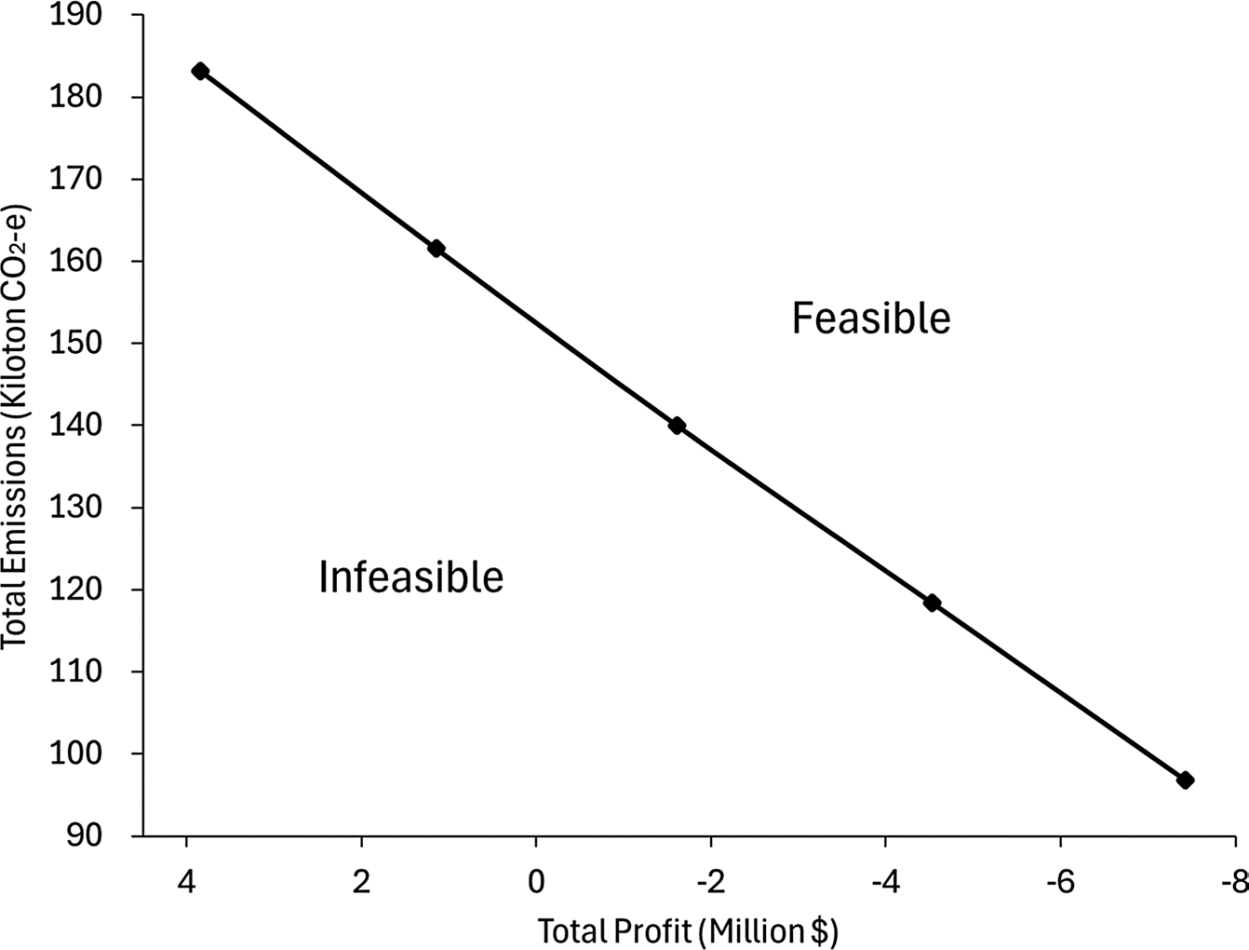
Pareto Frontier, generated based on five equally-spaced total emission (TE) values using the epsilon-constraint method

Figure 5 presents the Pareto Frontier of the feasible solutions, which represents the best trade-offs between the economic and environmental objectives. Decision-makers can select the solution that matches their priorities and limitations. It should be noted that while the proposed system configurations are based only on economic and environmental criteria, other political and social considerations play a significant role in directing the ideal system design. For example, the availability of land and utilities and the acceptance of local communities are crucial factors that can determine the feasibility and favorability of certain alternatives over others. However, these issues are beyond the scope of this paper, yet are interesting venues for future research.

### Sensitivity analysis

**Table 5:** Sensitivity analysis results corresponding to changes in the selling price of hydrogen ($$s_{k3},\,k\in K$$) in $/kg

Facility	TA1	TA2	TA3	Zones
$s_{k3}=2,\,k\in K$:	*TP = -3,484.13*			*TE = 145.61*
4	120,154	-	-	All
$s_{k3}=3,\,k\in K$:	*TP = 2,731.16*			*TE = 183.23*
3	-	-	23,004	1-9, 11
4	-	-	97,150	10, 12-29
$s_{k3}=4,\,k\in K$:	*TP = 12,163.30*			*TE = 183.23*
3	-	-	23,004	1-9, 11
4	-	-	97,150	10, 12-29

The table shows total profit (TP) in thousand $, total emissions (TE) in kiloton CO_2_e, and the processing capacities of installed TAs in thousand tons of MSW, along with the zones assigned to each facility

**Table 6:** Sensitivity analysis results corresponding to changes in the lower limit of the TA installed capacity ($\underline{q}_t,\, t\in T$) in tons of MSW/year

Facility	TA1	TA2	TA3	Zones
$\underline{q}_t=0,\, t\in T$:	*TP = -7,372.67*			*TE = 96.84*
1	-	9,916	-	10, 13
2	-	5,059		3, 7, 11, 12, 15
3	-	21,624	-	1, 2, 4-6, 8, 9
4	-	52,562		14, 16
5	-	7,849	-	18, 26
6	-	7,309	-	20, 22, 25
7	-	15,152	-	17, 19, 21, 23, 24, 27-29
$\underline{q}_t=5000,\, t\in T$:	*TP = -7,421.82*			*TE = 96.88*
1	-	9,916	-	10, 13
2	-	5,059		3, 7, 11, 12, 15
3	-	21,624	-	1, 2, 4-6, 8, 9
4	-	52,562		14, 16
5	-	7,849	-	18, 26
6	-	7,309	-	20, 22, 25
7	-	15,152	-	17, 19, 21, 23, 24, 27-29
$\underline{q}_t=10,000,\, t\in T$:	*TP = -7,404.77*			*TE = 96.90*
1	-	13,594	-	10, 12, 13, 15
3	-	23,004	-	1-9, 11,
4	-	52,562		14, 16,
6	-	10,291	-	20-22, 25, 26
7	-	20,704	-	17-19, 23, 24, 27-29
$\underline{q}_t=20,000,\, t\in T$:	*TP = -7,405.95*			*TE = 96.96*
1	-	20,658	-	10-15, 26
3	-	22,448	-	1-9
4	-	50,980	-	16, 20
7	-	26,068	-	17-19, 21-25, 27-29

The table shows total profit (TP) in thousand $, total emissions (TE) in kiloton CO_2_e, and the processing capacities of installed TAs in thousand tons of MSW, along with the zones assigned to each facility

Sensitivity of the optimal solution to changes in the values of critical model parameters is then investigated. When selecting these parameters, it is noted that most inputs used in the case study are based on real data, e.g., quantities, distances, shipping costs, and emissions, with only a handful of parameters subject to discretionary selection or significant uncertainty. Among those, the selling price of hydrogen ($s_{k3},\,k\in K$), the lower limit of the TA installed capacity ($\underline{q}_t,\, t\in T$), governmental subsidies, and the composition of MSW are considered in the sensitivity analysis.

**Fig. 6:**
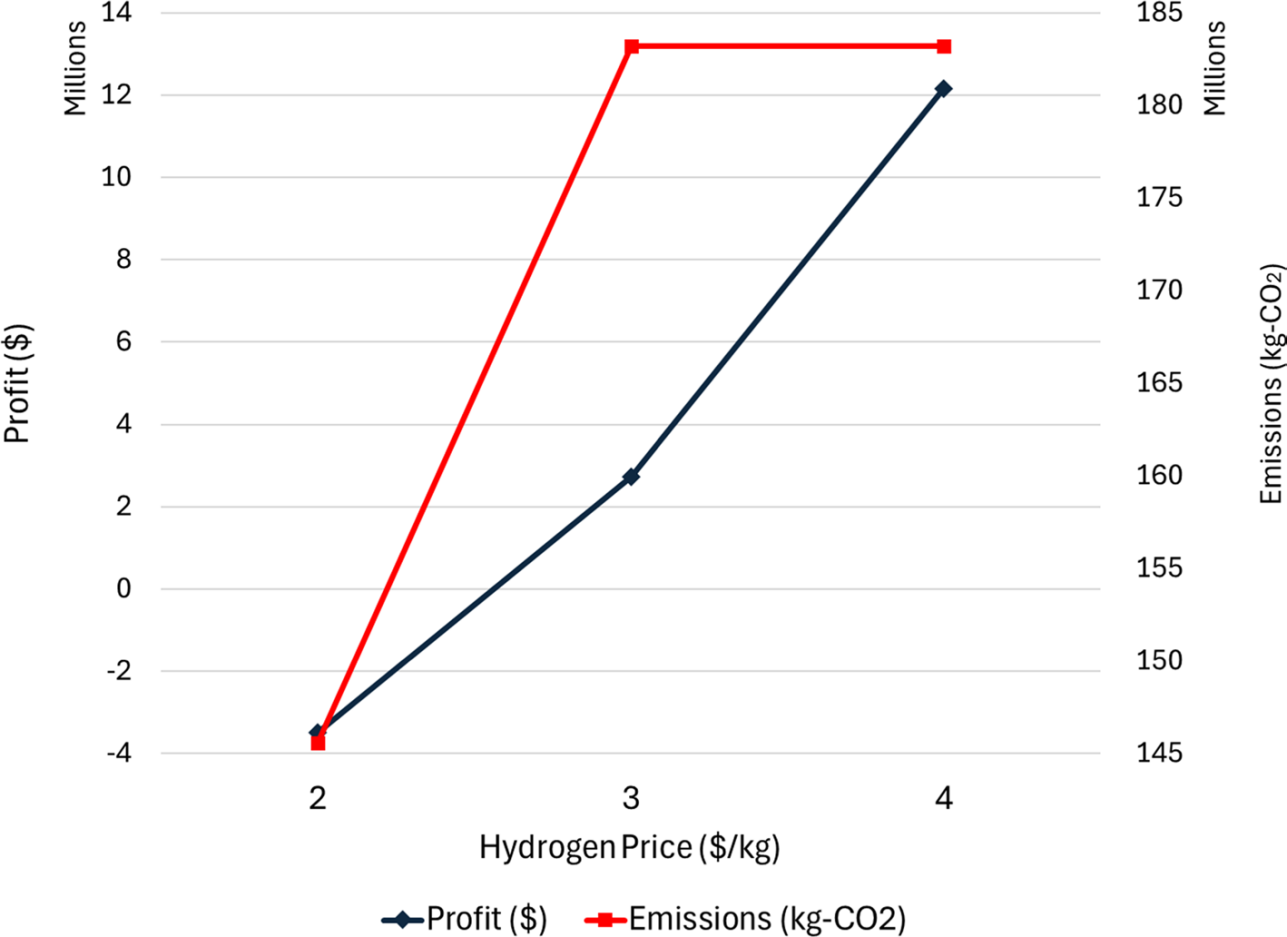
Sensitivity of profit and emissions to changes in the selling price of hydrogen

For the first factor, three hydrogen prices are tested: 2, 3, and 4 $/kg. Since this is an economic parameter, only the profit maximization problem is solved. The results depicted in Table 5 demonstrate that if the hydrogen selling price drops from 3 $/kg to 2 $/kg, TA3 (PAG with hydrogen separation) is no longer optimal, and the model selects TA1 (Incineration and CHP) instead. This is understandable, since TA3, albeit being highly energy-intensive, was economically feasible in the base case scenario due to the high price of hydrogen. So, when this price falls, it no longer becomes competitive. This technology switch results in higher centralization, by which only a single processing facility in Halifax is established to take advantage of the high economies of scale of TA1, and a moderate drop in TE (20.53%). In contrast, the positive profit of about $2.7M turns into a loss of about $3.5M with the hydrogen price decline and technology switching, demonstrating the crucial contribution of hydrogen extraction in the economics of MSWtE. On the other hand, when the selling price of hydrogen is increased to 4 $/kg, the MSWtE network structure remains unchanged. However, TP grew by around 245% due to the additional hydrogen revenues driven by its higher selling price, again demonstrating its importance as a revenue stream. Figure 6 illustrates the sensitivity of profit and emissions to changes in hydrogen price.

**Fig. 7:**
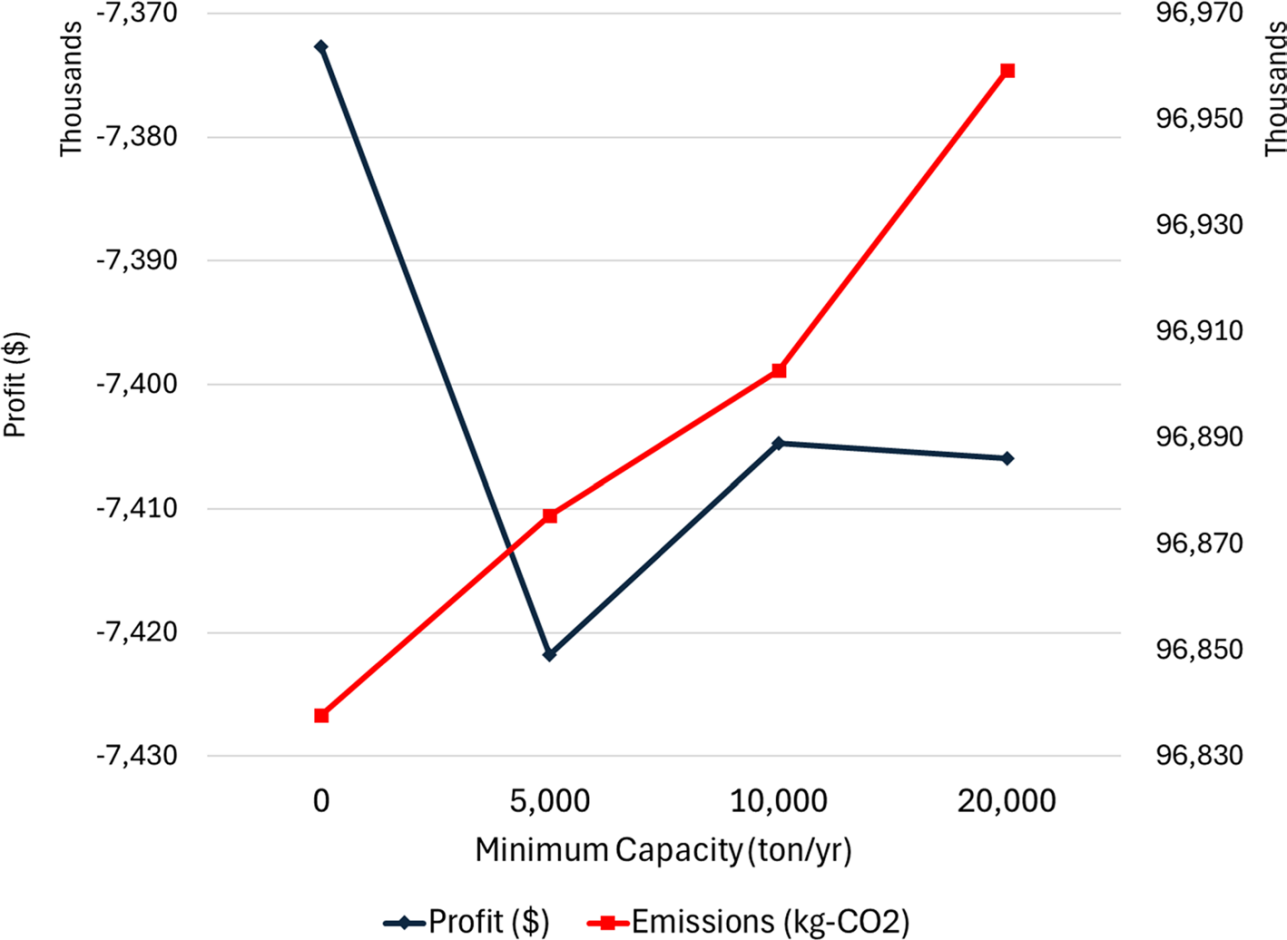
Sensitivity of profit and emissions to changes in the lower limit of technology capacity

In contrast, both TP and TE show very little sensitivity to the lower limit of technology capacity, as demonstrated in Table 6. Four values of $\underline{q}_t$ are tested: 0, 5,000, 10,000, and 20,000 ton/year. Since the lower limit was not restrictive for the profit maximization results in the base case scenario, only the emissions minimization problem is solved. As the minimum technology capacity constraint is further tightened (i.e., $\underline{q}_t$ is increased from 0 to 20,000), the objective value (TE) increased marginally from 96.84 to 96.96, a mere 0.12% change, while the total monetary loss increased by 0.45%. Yet, the notable change is in the number of facilities opened, which dropped from 7 to 4. As the minimum facility size becomes larger, fewer facilities are required to process the same quantity of MSW. Figure 7 illustrates the sensitivity of profit and emissions to changes in the lower limit of technology capacity.

Another potential source of revenue for the MSWtE system operator is the incentives introduced by governments to motivate green solutions that reduce GHG emissions. NS has introduced the Output-Based Pricing System (OBPS) in 2023, through which regulated facilities (e.g., large industrial or electricity generators) that exceed their emissions reduction targets receive “performance credit". Each credit represents one ton of GHG emissions reduced, can be sold, traded, or banked for the future, and has a predetermined value that is adjusted annually [44]. Other jurisdictions have similar schemes or alternative mechanisms, e.g., cap-and-trade programs. Hence, assuming that the MSWtE system operator is eligible for the OBPS, it is of interest to understand how such a scheme would affect the optimal system configuration and its corresponding economic and environmental performance.

The baseline used to calculate reductions in GHG emissions resulting from the proposed MSWtE framework is a traditional landfilling system, which represents the status quo. Based on the case study data, the emissions from transporting MSW from zones to the closest landfill are $$\sum \limits _{i\in I}\min \limits _{j\in J}\{b_{ij}\}$$, totaling 1,062,644 kg-CO_2_, whereas those attributed to landfilling are 660.89 $$ \sum\limits_{{i \in I}} {w_{i} } = $$ 79,408,789 kg-CO_2_. Furthermore, performance credits are earned by substituting conventionally generated energy products. Using the US average introduced in Section 3.2.3, each kWh of electricity corresponds to 0.368 kg-CO_2_. Similarly, to substitute the heat generated from MSW, it is assumed that heating oil will be used since it is the most common heating fuel in NS. The carbon intensity of Grade No. 2 heating oil is 0.226 kg-CO_2_ based on an emission factor of 2.753 kg-CO_2_/litre [45]. Finally, the full life-cycle footprint of grey hydrogen produced from Natural gas through Steam Methane Reforming (SMR) is 15.2 kg-CO_2_e/kg of hydrogen [46]. Several scenarios for the price of performance credits (i.e., the cost of carbon) are considered, ranging between 20 and 100 $/ton-CO_2_e. Incorporating these emissions and cost values in the cost minimization objective function and reoptimizing yields the optimal total revenue achievable, including from governmental subsidies.

Surprisingly, the system configuration exhibited no sensitivity to the price of carbon. For any monetary value of performance credits, the base case solution (without OBPS) remains optimal, with two facilities opening in locations 3 and 4, both using TA3. However, the total profit grew linearly as the price of carbon was increased, ranging from $2,731,156 in the base case to $6,792,723 when the price was set to $100/ton-CO_2_e. In all cases, the reduction in carbon emissions relative to the landfilling-based system was approximately 40,616 tons, thus the profit increased by this amount multiplied by the price of carbon.

A notable observation from the numerical results was that TA2, while being environmentally advantageous, was never selected for cost minimization. It is hypothesized that this poor economic performance is due to the fact that AD uses only organic waste (i.e., VS), which represents a small fraction of MSW, whereas the remainder is landfilled. Thus, the problem was solved with MSW containing higher fractions of VS (e.g., organics-rich or low-moisture waste) to see if this increases the economic appeal of TA2. Interestingly, even when the VS fraction is increased fourfold while holding all other parameters constant, the optimizer still favoured TA3 for cost minimization due to the high value of hydrogen generated from the PAG and hydrogen separation processes.

Before ending this section, it is worth discussing the computational aspects of the proposed model. All instances of the problems were solved in less than 2 s by Gurobi, demonstrating the ease with which such a long-term planning problem could be tackled. At first glance, this outcome is surprising, given that the problem is NP-hard in the strong sense, meaning that even its continuous relaxation is hard to solve due to the non-convex cost objective function, i.e., the terms $c_{t}(q_{jt})$. Besides the relatively small size of the instances ($|\mathcal {N}|=67$) and the sparsity of the node-arc incidence matrix, having single-variable concave functions means that modern solvers can readily apply tight piecewise-linear approximation techniques to these functions to deal with the non-linearity. However, for much larger instances, computational challenges are expected to arise, necessitating the resort to advanced relaxation, decomposition, and approximation techniques, e.g., branch-and-price or logic-based Benders decomposition. For example, the problem can be decomposed by potential facility location if the single assignment constraint (3) is relaxed or the binary variables are fixed, effectively splitting the model into a master problem and |*J*| (still difficult) subproblems. Another way is to use meta-heuristic techniques like Genetic Algorithms to reach high-quality (though not provably-optimal) solutions for large-scale instances of this intractable problem.

### Discussion and insights

Through the analysis of the results presented in this section, the following insights and policy recommendations can be drawn.

1. The issues of technology selection and the level of centralization/decentralization are intertwined through the economies of scale phenomenon in WtE technologies. While all the TAs considered in this work have sub-linear cost functions, this effect varies among them and may favor a given TA at a certain system scale and another at a different scale. For a province of about a million inhabitants, TA3 is the most economically viable, but for a much larger (e.g., 10 million) or smaller (e.g., 100,000) population, other TAs can prove more economical. Likewise, economies of scale strongly favor centralization, as its absence in the emission-minimization scenario led to a totally decentralized system. However, this effect is scale-dependent since the system tries to balance the cost savings achieved via consolidation with the additional shipping costs incurred. Other factors can also affect the level of centralization, such as the lower limit of the TA installed capacity (e.g., only a single minimum-capacity facility is sufficient to process the entire waste quantity) or social and political considerations (e.g., the desire to create jobs in remote communities or to keep facilities away from urban areas). These issues must be considered carefully before determining the optimal level of (de-)centralization.
2. Among the uncertain model inputs tested in the sensitivity analysis, the hydrogen price was the most influential and the primary driver for exclusively selecting TA3 in the profit maximization scenario. If the price of hydrogen declines, as expected in the future due to technological advances and production scale-ups, this can have a profound impact on the optimal design and profit of the MSWtE system. Hence, decision-makers must be mindful of the high sensitivity of their expected outcomes to this specific factor; today’s optimal design might not remain so in a few years, underscoring the importance of a long-term vision. A secondary effect is that the results might become more sensitive to other factors, e.g., waste composition, as the dominant effect of hydrogen price dwindles. For instance, with an organic-rich waste, TA2 can become more economically viable if the lucrative revenue stream from selling hydrogen shrinks. Thus, the sensitivity analysis results, while valid for the considered case study, must be taken with caution in other contexts and not over-generalized.
3. The OBPS framework currently implemented in NS proved insufficient to motivate profit-seeking WtE investors to adopt the most environmentally-conscious TA, namely TA2, since it did not adequately compensate for the missed hydrogen selling opportunity. Under current market conditions, the government needs to either increase the monetary value of performance credits or impose strict carbon caps to incentivise this shift. However, as noted in the previous point, these changes might be unnecessary if hydrogen becomes less expensive. The dynamic relationship between the *price of carbon* and the *price of hydrogen* is an intriguing phenomenon that deserves attention from policymakers.
4. Proximity of facilities to the sources of waste and the sinks of energy products is crucial for economically- and environmentally-viable systems. Numerical results showed that, regardless of the number of open facilities, they were often centrally located close to their assigned supply and demand nodes. This was particularly the case when the energy product was hydrogen, which is costly to ship, thus the facilities are placed in the closest potential locations to the two export ports.

## Conclusion

A bi-objective strategic framework is proposed to simultaneously optimize the facility location and capacity, zone assignment, technology selection, and flow of waste and energy products in an MSWtE network. In the course of developing the mathematical model, the cost, emission, and conversion functions of three technology alternatives, namely incineration with a steam turbine, AD with CHP, and PAG with hydrogen separation, are derived in terms of the MSW processing capacity. The proposed framework is tested on a realistic case study in NS, and the results are analyzed.

Numerical results show that the optimal MSWtE system configuration and its economic and environmental performance greatly depend on the objective sought. A profit maximization objective favors a centralized system that utilizes plasma arc gasification and generates hydrogen to take advantage of the economies of scale and the lucrative hydrogen export opportunity. Conversely, if the priority is to minimize the carbon footprint of the system, anaerobic digestion of food waste and landfilling the remainder is the best option, with a decentralized system that minimizes shipping-related emissions. Sensitivity analyses demonstrated that the selling price of hydrogen plays an impactful role, whereas the minimum allowable capacity makes little difference in the total profit and GHG emissions. When government subsidies, in the form of performance credits, are considered, they significantly increase profit but do not affect the optimal system design. Likewise, the optimal system design proved robust against some changes in the composition of MSW. In particular, increasing the fraction of volatile solids in the waste failed to make TA2 more economically attractive than TA3 due to the lucrative hydrogen export opportunity presented by the latter.

Besides its methodological contributions, in the form of the integrated MSWtE design model and the derivation of cost, emission, and conversion functions for multiple TAs, this study offers useful managerial insights for partitioners and policymakers. First, it demonstrates that with the careful selection of WtE technologies, facility locations, and capacities, MSW can be turned from an economic burden into a profit opportunity by delivering high-value downstream products like hydrogen to markets. This profit, while being quite sensitive to the price of hydrogen, can be leveraged via governmental subsidies or by selling carbon credit certificates. The numerical results also show that economies of scale affect the optimal system configuration, leading to a centralized system consisting of a few large processing facilities in the case of profit maximization, given the concave cost functions, and to a decentralized system when GHG emissions are to be minimized, given the linear emission functions. Thus, ignoring this effect and designing the system based on elementary calculations of average costs can lead to grossly inaccurate results and poor designs. Moreover, when selecting the locations of MSWtE facilities, it seems that proximity to the demand nodes plays a crucial role, especially for downstream products that are expensive to transport, like hydrogen. The two locations used for installing TA3 in the profit maximization scenario are those closest to the hydrogen export ports. Another important observation is the clear tradeoff between economic and environmental objectives, apparent in the Pareto frontier. However, results also show that it is possible to find a balanced design that achieves an economic break-even by using realistic emission reduction targets in the bi-objective model. In the case of NS, this target is about 1.321 ton-CO_2_e per ton of MSW.

Although many of the study inputs are universal and thus usable across various geographical and regulatory contexts, e.g., the conversion and emission functions of technologies, some are specific to the case study, especially technology and shipping costs. Thus, the findings and recommendations must be taken cautiously when the implementation context is vastly different. Fortunately, the proposed optimization framework is quite generic and can be applied as-is or with minor changes to cases with different assumptions or inputs. It is also important to note that while the cost, emission, and conversion functions were derived meticulously based on the best publicly available data sources, albeit sometimes a bit dated, they are subject to change over time given the rapid advancements in WtE technologies, so they must be revised and calibrated regularly. Another limitation of the study is its somewhat narrow focus on profit and GHG emissions as the only criteria for assessing alternative TAs and system configurations. On the other hand, it ignores social and political aspects such as the social acceptance of MSWtE technologies, their integrability with existing market and society structures (e.g., energy companies and municipalities), the issues of land ownership and usage, the job opportunities created or lost by the new system, and energy access and security for the communities that rely on the energy products delivered by the proposed system; see, e.g., [47, 48], for discussions on the social impacts of WtE technologies. These issues, and others, must be considered before making significant changes in the existing waste management structure.

The current research can be extended by considering additional WtE technologies such as gasification and pyrolysis, or combinations of technologies (e.g., AD for food waste and gasification for other waste types). Furthermore, the opportunity to extract and sell other liquid and solid downstream products, such as bio-oil and bio-char, can be considered. Another venue for future research is to include other objectives and constraints related to the social impacts of WtE technologies in the mathematical formulation, or to consider a competitive market situation in which multiple agents offer alternative solutions and aim to maximize their gains, along with a market regulator setting the rules and determining the payoffs. Finally, incorporating the routing of waste-collection trucks as a decision to be made indigenously by the optimization model is another direction for extension.

## Data Availability

All data and codes used in this study are available on request from the authors.

## References

[1] Gautam M, Agrawal M. In: Muthu SS. (ed.) Greenhouse gas emissions from municipal solid waste management: a review of global scenario, pp. 123–160. 2021;Springer, Singapore. 10.1007/978-981-15-9577-6_5

[2] The World Bank: Trends in Solid Waste Management. Available: https://datatopics.worldbank.org/what-a-waste/trendsinsolidwastemanagement.html. [Accessed 10 June 2025]

[3] Eisted R, Larsen A, Christensen T. Collection, transfer and transport of waste: accounting of greenhouse gases and global warming contribution. Waste Manag Res J Sustain Circ Econ. 2009;27(2):738–45. 10.1177/0734242X09347796. 10.1177/0734242X09347796 19808734

[4] The U.S. Energy Information Administration: Renewable energy explained. Available: www.eia.gov/energyexplained/renewable-sources [Accessed 31 July 2025]

[5] Saif A, Elhedhli S. A Lagrangian heuristic for concave cost facility location problems: the plant location and technology acquisition problem. Optim Lett. 2016;10:1087–100. 10.1007/s11590-016-0998-4.

[6] Li T, Deng S, Lu C, Wang Y, Liao H. Optimization of green vehicle paths considering the impact of carbon emissions: a case study of municipal solid waste collection and transportation. Sustainability. 2023;15(22):16128. 10.3390/su152216128.

[7] Neto ABPS, Simões CL, Simoes R. Optimization of municipal solid waste collection system: systematic review with bibliometric literature analysis. J Mater Cycles Waste Manage. 2024;26:1906–17. 10.1007/s10163-024-01966-y.

[8] Boresta M, Croella AL, Gentile C, Palagi L, Pinto DM, Stecca G, et al. Optimal network design for municipal waste management: application to the metropolitan city of rome. Logistics. 2024;8(3):79. 10.3390/logistics8030079.

[9] Silva MG, Przybysz AL, Piekarski CM. Location as a key factor for waste to energy plants. J Clean Prod. 2022;379:134386. 10.1016/j.jclepro.2022.134386.

[10] Mostafavi Sani M, Afshari H, Saif A. A robust framework for waste-to-energy technology selection: a case study in Nova Scotia, Canada. Energy Convers Manage. 2023;284:116965. 10.1016/j.enconman.2023.116965.

[11] Pluskal J, Šomplák R, Hrabec D, Nevrlý V, Hvattum LM. Optimal location and operation of waste-to-energy plants when future waste composition is uncertain. Oper Res Int J. 2022;22:5765–90. 10.1007/s12351-022-00718-w.

[12] Esfilar R, Bagheri M, Golestani B. Technoeconomic feasibility review of hybrid waste to energy system in the campus: a case study for the university of victoria. Renew Sustain Energy Rev. 2021;146:111190. 10.1016/j.rser.2021.111190.

[13] Armoo EA, Narra S, Mohammed M, Boahemaa B, Beguedou E, Kemausuor F, et al. Hybrid waste-to-energy solutions within a circular economy framework directed towards sustainable urban waste management in ghana. Sustainability. 2024;16(12):4976. 10.3390/su16124976.

[14] Inayat A, Dafalla M, Asaad S, Jamil F, Al-Haj L, Shah FM, et al. Sustainable energy production from waste: a review of hybrid approaches combining anaerobic digestion and gasification. Int J Energy Res. 2025;2025(1):6644084. 10.1155/er/6644084.

[15] Gonçalves Mollica GJ, Cerda Balcazar JG, Alves Dias R, Perrella Balestieri JA. Sustainable waste management solutions: optimization of hybrid and gasification waste-to-energy plants. Energy. 2024;306:132549. 10.1016/j.energy.2024.132549.

[16] Saatchi P, Salamian F, Manavizadeh N, Rabbani M. A sustainable network design for municipal solid waste management considering waste-to-energy conversion under uncertainty. Eng Optim. 2025;57(9):2505–28. 10.1080/0305215X.2024.2408478.

[17] Saif A, Masri H. Waste-to-energy technology selection and capacity planning in a multi-facility waste management system. In: Proc. the 11th international conference on energy, sustainability and climate crisis (ESCC 2024), Corfu, Greece, pp. 174–180, 2024. http://escc.uth.gr/escc-series/escc-2024

[18] Rahman IU, Mohammed HJ, Bamasag A. An exploration of recent waste-to-energy advancements for optimal solid waste management. Discovry Chem Eng. 2025;5:7. 10.1007/s43938-025-00079-8.

[19] Pilarska AA, Kulupa T, Kubiak A, Wolna-Maruwka A, Pilarski K, Niewiadomska A. Anaerobic digestion of food waste–a short review. Energies. 2023;16(15):5742. 10.3390/en16155742.

[20] Byun Y, Cho M., Hwang S-M, Chung J. Thermal plasma gasification of municipal solid waste (msw). In: Yun Y. (ed.) Gasification for Practical Applications. IntechOpen, Rijeka, 2012. Chap. 7. 10.5772/48537

[21] Tehrani F, Haghi E. Techno-economic assessment of municipal solid waste incineration plant—case study of Tehran, Iran. In: In Proc. The First sustainable development conference of engineering systems in energy, water and environment, Tehran, pp. 1–4, 2011. https://api.semanticscholar.org/CorpusID:33973442

[22] Lee H, Yi S-M, Holsen TM, Seo Y-S, Choi E. Estimation of co2 emissions from waste incinerators: comparison of three methods. Waste Manage. 2018;73:247–55. 10.1016/j.wasman.2017.11.055. 10.1016/j.wasman.2017.11.055 29221870

[23] Abuadala A, Dincer I, Naterer GF. Exergy analysis of hydrogen production from biomass gasification. Int J Hydrogen Energy. 2010;35(10):4981–90. 10.1016/j.ijhydene.2009.08.025.

[24] Ibikunle RA, Titiladunayo IF, Akinnuli BO, Dahunsi SO, Olayanju TMA. Estimation of power generation from municipal solid wastes: a case study of Ilorin metropolis, Nigeria. Energy Rep. 2019;5:126–35. 10.1016/j.egyr.2019.01.005.

[25] Arsova L. Anaerobic digestion of food waste: Current status, problems and an alternative product. Master’s thesis, Columbia University, 2010. https://api.semanticscholar.org/CorpusID:16728263

[26] Vasco-Correa J, Khanal S, Manandhar A, Shah A. Anaerobic digestion for bioenergy production: global status, environmental and techno-economic implications, and government policies. Biores Technol. 2018;247:1015–26. 10.1016/j.biortech.2017.09.004. 10.1016/j.biortech.2017.09.004 28918346

[27] Li Y, Park SY, Zhu J. Solid-state anaerobic digestion for methane production from organic waste. Renew Sustain Energy Rev. 2011;15(1):821–6. 10.1016/j.rser.2010.07.042.

[28] Lantz M. The economic performance of combined heat and power from biogas produced from manure in Sweden—a comparison of different chp technologies. Appl Energy. 2012;98:502–11. 10.1016/j.apenergy.2012.04.015.

[29] Eder B, Schulz H. Biogas-Praxis: Grundlagen, Planung, Anlagenbau, Beispiele, Wirtschaftlichkeit. Ökobuch magnum. Ökobuch, Berlin, Germany, 2007. https://oekobuch.de/buecher/biogas-praxis

[30] Murphy JD, McKeogh E. Technical, economic and environmental analysis of energy production from municipal solid waste. Renew Energy. 2004;29(7):1043–57. 10.1016/j.renene.2003.12.002.

[31] Mar KA, Unger C, Walderdorff L, Butler T. Beyond co2 equivalence: the impacts of methane on climate, ecosystems, and health. Environ Sci Policy. 2022;134:127–36. 10.1016/j.envsci.2022.03.027.

[32] Manfredi S, Tonini D, Christensen TH, Scharff H. Landfilling of waste: accounting of greenhouse gases and global warming contributions. Waste Manag Res. 2009;27(8):825–36. 10.1177/0734242X09348529. 19808732 10.1177/0734242X09348529

[33] Chanthakett A, Arif MT, Khan MMK, Oo AMT. Performance assessment of gasification reactors for sustainable management of municipal solid waste. J Environ Manage. 2021;291:112661. 10.1016/j.jenvman.2021.112661. 33962284 10.1016/j.jenvman.2021.112661

[34] Arena U. Process and technological aspects of municipal solid waste gasification. A review. Waste Manage. 2012;32(4):625–39. 10.1016/j.wasman.2011.09.025. 10.1016/j.wasman.2011.09.025 22035903

[35] Ramos A, Berzosa J, Espí J, Clarens F, Rouboa A. Life cycle costing for plasma gasification of municipal solid waste: A socio-economic approach. Energy Convers Manage. 2020;209:112508. 10.1016/j.enconman.2020.112508.

[36] Ducharme C. Technical and economic analysis of plasma-assisted waste-to-energy processes. Master’s thesis, Columbia University, 2010. https://api.semanticscholar.org/CorpusID:18205264

[37] Nguyen TTT, Wilson BG. Fuel consumption estimation for kerbside municipal solid waste (msw) collection activities. Waste Manage Res. 2010;28(4):289–97. 10.1177/0734242X09337656. 10.1177/0734242X09337656 19723822

[38] Control Centre Operations: Interconnection feasibility study report gip-ir703-feas-r0. Technical report, Nova Scotia Power Inc. 2024. Available: https://www.nspower.ca/docs/default-source/pdf-to-upload/gip-ir703-feas-r0-report-(300mw-poi-on-l-7005).pdf?sfvrsn=917e6993_3 [Accessed 26 March 2026]

[39] Transmission and Distribution Engineering Department: Feasibility study infra-structure report for ir#56 - establishing a 69 kv system interconnection for a new 34 mw wind powered generating facility at pugwash, nova scotia. Technical report, Nova Scotia Power Inc. 2010. Available: https://www.nspower.ca/docs/default-source/pdf-to-upload/ir56-fac-summary(1).pdf?sfvrsn=e0092fec_0. [Accessed 26 March 2026]

[40] Sohrabikhah S, Hughes L. A performance evaluation and feasibility study of mine thermal energy storage in glace bay, nova scotia. Energies. 2025;18(17):4780. 10.3390/en18174780.

[41] Natural Resources Canada: Furnace Oil Prices. Available at: https://natural-resources.canada.ca/domestic-international-markets/furnace-oil-prices. [Accessed 26 March 2026]

[42] NS Department of Environment and Climate Change: Municipal Solid Waste Disposal Totals. Available: https://data.novascotia.ca/Environment-and-Energy/Municipal-Solid-Waste-Disposal-Totals/mp68-kdz7/aboutdata [Accessed 10 June 2025]

[43] NS Department of Environment and Climate Change: Municipal Solid Waste Disposal Sites. Available: https://novascotia.ca/nse/waste/solidwastedisposal.asp [Accessed 10 June 2025]

[44] Province of Nova Scotia: Output-Based Pricing System Reporting and Compliance Regulations, N.S. Reg. 24/2024. Environment Act, S.N.S. 1994-95, c. 1. Effective January 1, 2023; consolidation current to August 3, 2024, 2024. https://novascotia.ca/just/regulations/regs/envoutputcompliance.htm

[45] Environment and Climate Change Canada: National inventory report 1990–2022: Greenhouse gas sources and sinks in Canada. Annual report, Government of Canada, 2024. Canada’s official submission to the United Nations Framework Convention on Climate Change (UNFCCC). https://publications.gc.ca/site/eng/9.506002/publication.html

[46] Sovacool BK, Griffiths S, Herman K, Iskandarova M. A critical meta-survey of the lifecycle greenhouse gas emissions of hydrogen energy systems. Int J Hydrogen Energy. 2026;57:660–78. 10.1016/j.ijhydene.2024.01.034.

[47] Michael T. 2 - environmental and social impacts of waste to energy (wte) conversion plants. In: Klinghoffer NB, Castaldi MJ, editors. Waste to Energy Conversion Technology. Woodhead Publishing Series in Energy. Woodhead Publishing; 2013. p. 15–28. 10.1533/9780857096364.1.15.

[48] Agaton CB, Santos MJA, Calvelo JAS. Progress in socioeconomic and environmental impacts of waste-to-energy technologies in a circular economy: a systematic review. J Human Ecol Sustain. 2025;3(1):6. 10.56237/jhes-25-018.

